# Racial Disparities in Plasma Cell Leukemia Outcomes Among Hospitalized Patients in the United States

**DOI:** 10.46989/001c.87755

**Published:** 2023-09-08

**Authors:** Cindy Wu, Deepa Dongarwar, Samer Al Hadidi

**Affiliations:** 1 Baylor College of Medicine, Department of Internal Medicine, Houston, TX, USA; 2 McGovern School of Medicine, The University of Texas at Houston, Houston, TX, USA; 3 Myeloma Center, Winthrop P. Rockefeller Cancer Institute University of Arkansas for Medical Sciences, Little Rock, AR, USA

**Keywords:** African American, Black persons, disparity, multiple myeloma, plasma cell leukemia

## Abstract

Plasma cell leukemia (PCL) is a rare, aggressive subtype of multiple myeloma (MM) with a poor prognosis. Prior studies have shown that racial disparities affect MM patients in various ways, which may affect patients’ outcomes. In this study, we aimed to investigate racial differences in hospitalization outcomes for PCL using Nationwide Inpatient Sample data. Overall, hospitalization rates for PCL tended to decrease over the past decade. Among hospitalized patients with a primary diagnosis of PCL, there was no statistically significant association between race/ethnicity and hospitalization rates, between NH-White patients and NH-Black patients (OR 1.94; 95%CI 0.3-3.54, p 0.95), and Hispanic patients (OR 0.47; 95% CI 0.05-4.23, p 0.5). Additionally, there was no significant association between race/ethnicity and inpatient mortality. The overall lower incidence of PCL, more significant disease burden, and poor prognosis across all groups may contribute to our findings. With increasing evidence that PCL is cytogenetically distinct from MM, more investigation into biological and sociodemographic factors that affect healthcare utilization and treatment outcomes should be carried out.

## Introduction

Plasma cell leukemia (PCL) is a rare subtype of multiple myeloma (MM) with a poor prognosis. While primary PCL was traditionally classified as “de novo” and presenting in patients without a previous diagnosis of MM,^1^ recent updates by the International Myeloma Working Group have now defined primary PCL by the presence of ≥ 5% circulating plasma cells in the peripheral blood in patients diagnosed with symptomatic MM, based on evidence that patients in this category had similar adverse outcomes with previously defined PCL (≥ 20% circulating plasma cells in peripheral blood).^2^ Racial disparities have previously been described for MM regarding disease prevalence, treatment outcomes, and mortality. Non-Hispanic Black and Hispanic American patients have a higher incidence of MM, as well as a higher prevalence of monoclonal gammopathy of undetermined significance (MGUS), and Black patients tend to be diagnosed with both MM and MGUS at younger ages, with a previous study showing that Black patients have a 4-year earlier age of onset of MM compared to White patients.^3,4^ Additionally, in the National Health and Nutritional Examination Survey (NHANES) III, the highest disparity prevalence of MGUS between Black and White patients was seen in the 40-49 age group.^5^ A previous study also showed that Black patients have a higher prevalence of MM-related hospitalizations than non-Hispanic White patients and Hispanic patients had higher MM-related in-hospital mortality.^6^ Despite this, non-Hispanic Black and Hispanic patients continue to have a lower rate of enrollment in clinical trials utilizing chimeric antigen receptor T-cell therapy (CART-T) and other novel treatment agents.^7,8^ Disparities in treatment outcomes and mortality for MM are likely related to socioeconomic and genomic factors^4^ However, limited data exists for racial/ethnic differences in patients with PCL. We aimed to investigate ethnic/racial differences in hospitalization outcomes for PCL.

## Materials and Methods

All data were derived from the 2010-2019 Nationwide Inpatient Sample (NIS), the largest all-payer hospitalization database from the Healthcare Cost and Utilization Project (HCUP). It captures hospital discharge data from the 48 US states and the District of Columbia, covering approximately a fifth of all US hospitalizations, excluding rehabilitation hospitals. International Classification of Disease Clinical Modification (ICD-CM) billing codes from the ninth revision (ICD-9-CM, from 2010 to the third quarter of 2015) and tenth revision (ICD-10-CM, from the fourth quarter of 2015 to 2019) were utilized to identify medical diagnoses and procedures. Socio-demographics provided in the data set included age, sex (male, female), race/ethnicity (Hispanic, Non-Hispanic (NH) Black, NH-White, NH-Other, or missing), primary hospital payer (Medicare, Medicaid, private insurance, self-pay, other), median zip code income quartile, disposition (routine, transfer, died, discharged against medical advice [DAMA] and other), hospital bed size (small, medium, large), hospital location and teaching status (rural, urban non-teaching, urban teaching), and geographic location within the United States (Northeast, South, Midwest, South).

All hospitalizations for patients 18 years or older adults with a discharge diagnosis of plasma cell leukemia as a primary or secondary diagnosis were included. The ICD-9-CM codes used for PCL were 203.1.x, whereas ICD-10-CM codes were C90.1x. Descriptive statistics were used to describe our cohort and to estimate prevalence per 100,000 hospitalizations by age, sex, race/ethnicity, disposition, zip code income quartile, primary payer, hospital region, hospital bed, hospital location, and teaching status, in the overall study population and in those with plasma cell leukemia. Bivariate statistical significance was assessed using Pearson’s chi-squared test. Joinpoint regression was used to analyze temporal trends for plasma cell leukemia from 2010-2019, stratified by race and ethnicity.

Average Annual Percentage Change (AAPC) and 95% confidence intervals were used to represent the rate of change in hospitalizations. Survey logistic regression models were built to assess the association between sociodemographic factors and plasma cell leukemia hospitalizations. In each model, we adjusted for the following covariates: age, sex, race/ethnicity, zip code income quartile, primary payer, hospital region, hospital bed size, and teaching status. For all regression models, the reference categories were set as male (sex), young age (18-39 for this study), NH-White (for race/ethnicity), lowest income quartile, private insurance payer, routine disposition, Northeast hospital region, small bed size, rural non-teaching location. All analyses were conducted using R (version 3∙6∙1), RStudio (Version 1∙2∙5001), or join point Regression Program, version 4.7.0.0 (National Cancer Institute). All tests of statistical significance were two-tailed with alpha set at 5%. Given that the data is publicly available, the study was considered exempt by the Institution Review Board at the University of Arkansas for Medical Sciences.

## Results

Between 2010 and 2019, there were 14303 hospitalizations for patients with a primary diagnosis of PCL within the NIS database. Of this cohort, 53.6% were male. Most patients (83%) were between the ages of 40 and 79, with 6.8% ranging from 18-39 years and 10.2% at 80 years or older. The racial/ethnic distribution of this cohort includes 7.8% Hispanic, 22.2% NH-Black, 59.4% Non-Hispanic (NH) White, 4.6% NH-Other, and 5.9% with missing demographic information for race/ethnicity. Additional demographic characteristics are summarized in **Table 1**.

**Table 1.** Demographic characteristics of patients with plasma cell leukemia

	**Total**		**Plasma cell leukemia**		**Prevalence per 100,000 hospitalizations**	**p-value**
	**N=304480139**	**%=100**	**N=14303**	**%=100**		
**Gender**						<0.01
Male	126276359	41.47%	7669	53.62%	6.0731875	
Female	178096256	58.49%	6629	46.35%	3.7221445	
Missing	107524	0.04%	─	─	─	
**Age**						<0.01
18-39 years	74009571	24.31%	973	6.80%	1.3146948	
40-59 years	75799239	24.89%	4804	33.59%	6.3377945	
60-79 years	103746143	34.07%	7061	49.37%	6.8060362	
80+ years	50925186	16.73%	1465	10.24%	2.8767691	
**Race/Ethnicity**						<0.01
NH-White	195552023	64.22%	8495	59.39%	4.3441126	
NH-Black	43501544	14.29%	3179	22.23%	7.3077866	
Hispanic	31118963	10.22%	1122	7.84%	3.6055186	
NH-Others	17724049	5.82%	662	4.63%	3.7350382	
Missing	16583559	5.45%	844	5.90%	5.089378	
**Disposition**						<0.01
Routine	197456813	64.85%	6905	48.28%	3.4969672	
Transfer	55405931	18.20%	3157	22.07%	5.6979459	
Died	6661290	2.19%	1484	10.38%	22.277967	
DAMA	4125114	1.35%	75	0.52%	1.8181316	
Other	40639779	13.35%	2676	18.71%	6.5846815	
Missing	191211	0.06%	─	─	─	
**Zip code Income quartile**						<0.01
Lowest quartile	89832445	29.50%	3508	24.53%	3.9050479	
Second quartile	77223124	25.36%	3449	24.11%	4.4662788	
Third quartile	71316441	23.42%	3589	25.09%	5.0325001	
Highest quartile	59779208	19.63%	3467	24.24%	5.7996754	
Missing	6328919	2.08%	288	2.01%	4.5505401	
**Primary Payer**						<0.01
Medicare	142155872	46.69%	6372	44.55%	4.4824037	
Medicaid	52296258	17.18%	1629	11.39%	3.1149456	
Private Insurance	84605886	27.79%	5284	36.94%	6.2454284	
Self-Pay	24860100	8.16%	973	6.80%	3.9139022	
Other	562023	0.18%	45	0.31%	8.0067898	
**Hospital Region**						<0.01
Northeast	58223641	19.12%	2802	19.59%	4.8124781	
Midwest	68791079	22.59%	3120	21.81%	4.5354718	
South	118974593	39.07%	5474	38.27%	4.6009823	
West	58490825	19.21%	2906	20.32%	4.9683006	
**Hospital Bed Size**						<0.01
Small	52146784	17.13%	1573	11.00%	3.0164852	
Medium	83884697	27.55%	2595	18.14%	3.0935321	
Large	167672304	55.07%	10099	70.61%	6.0230579	
Missing	776354	0.25%	36	0.25%	4.6370599	
**Hospital Location and Teaching Status**						<0.01
Rural	31330118	10.29%	683	4.78%	2.1800109	
Urban non-teaching	92357695	30.33%	2387	16.69%	2.5845166	
Urban teaching	180015971	59.12%	11196	78.28%	6.2194482	
Missing	776354	0.25%	36	0.25%	4.6370599	

**Figure 1.**
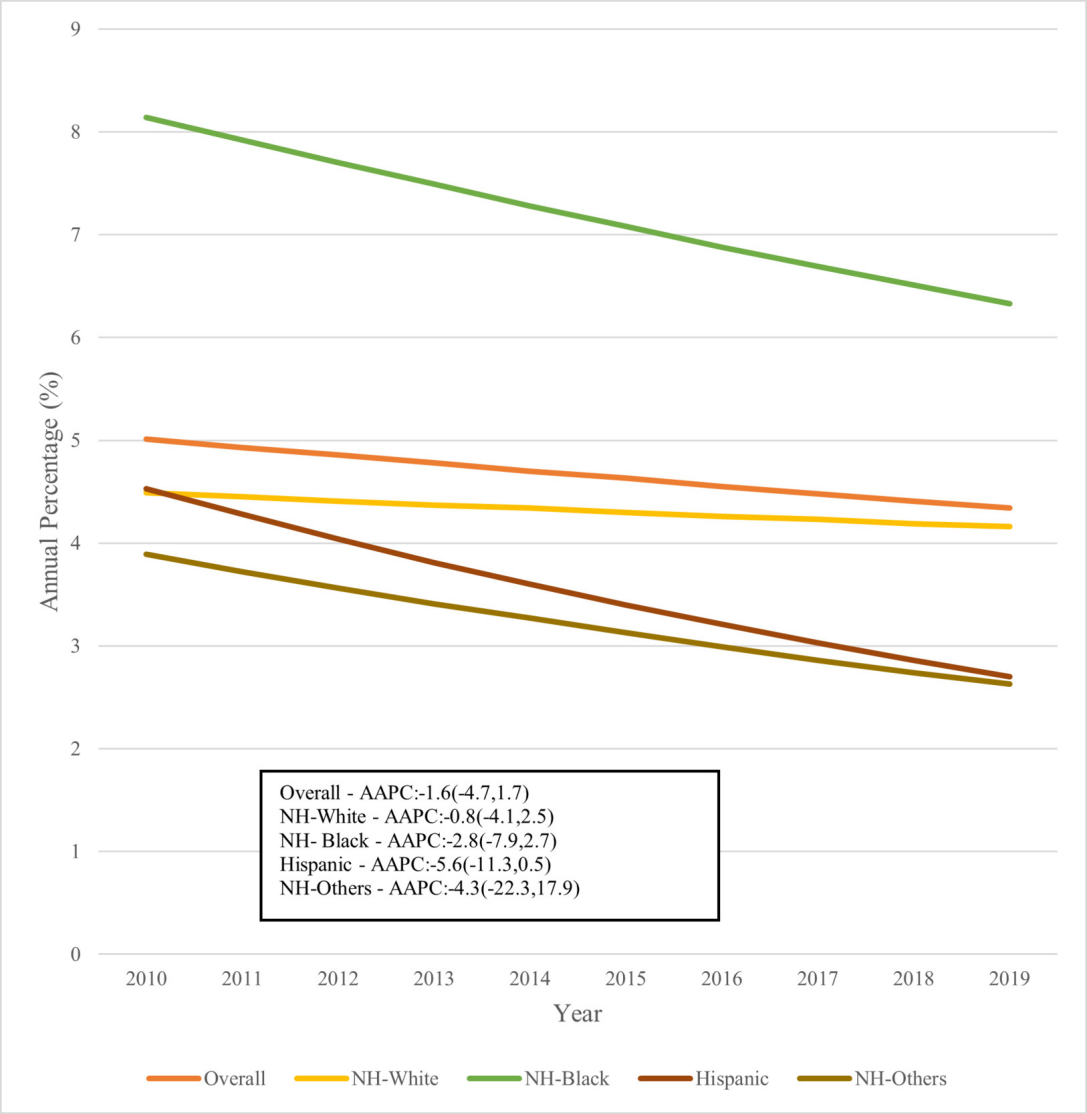
Trends in plasma cell leukemia hospitalizations by race/ethnicity - 2010-2019

Over this 10-year period, there was a tendency towards lower hospitalization rates across the entire cohort, with an AAPC of -1.6 in each ethnic/racial category, as shown in **Figure 1**.

Among hospitalized patients with a primary diagnosis of PCL, there was no statistically significant association between race/ethnicity and hospitalization rates, between NH-White and, Hispanic (OR 0.47; 95% CI 0.05-4.23, p 0.5), NH-Black (OR 1.94; 95%CI 0.3-3.54, p 0.95), and NH-Others (OR 1.67, CI 0.47-5.89, p 0.42). Additionally, there was no significant association between gender and hospitalization rates (OR 1.52; 95% CI 0.67-3.42, p 0.32). No significant association was seen between age and hospitalization rates between the 18-39 years age group and 40-59 years (OR 3.78; CI 0.77-18.52, p 0.1), 60-79 years (OR 2.97; CI 0.65-13.49, p 0.16), and 80+ years (OR 1.22; CI 0.2-7.29, p 0.83). In this cohort, hospitalization rates were not significantly associated with death (OR 1.85; CI 0.22-15.41, p 0.57). Other factors examined include zip code income quartile, hospital region, hospital location and teaching status, hospital bed size, and primary payer/insurance status, none of which had a significant association with PCL hospitalizations, as listed in **Table 2**.

**Table 2.** Factors associated with plasma cell leukemia hospitalizations

	**OR (95% CI)**	**p-values**
**Gender**		
Male	reference	
Female	1.52(0.67-3.42)	0.32
**Age**		
18-39 years	reference	
40-59 years	3.78(0.77-18.52)	0.1
60-79 years	2.97(0.65-13.49)	0.16
80+ years	1.22(0.2-7.29)	0.83
**Race/Ethnicity**		
NH-White	reference	
NH-Black	1.04(0.3-3.54)	0.95
Hispanic	0.47(0.05-4.23)	0.5
NH-Others	1.67(0.47-5.89)	0.42
**Disposition**		
Routine	reference	
Transfer	1.17(0.45-3.05)	0.75
Died	1.85(0.22-15.41)	0.57
DAMA	─	─
Other	1.79(0.71-4.51)	0.22
Missing		
**Zip code Income quartile**		
Lowest quartile	reference	
Second quartile	1.06(0.28-4.01)	0.93
Third quartile	1.36(0.42-4.45)	0.61
Highest quartile	1.77(0.53-5.88)	0.35
**Primary Payer**		
Medicare	reference	
Medicaid	0.42(0.1-1.81)	0.24
Private Insurance	0.82(0.38-1.77)	0.62
Self-Pay	─	─
**Hospital Region**		
Northeast	reference	
Midwest	0.46(0.14-1.44)	0.18
South	0.62(0.24-1.64)	0.34
West	0.53(0.17-1.71)	0.29
**Hospital Bed Size**		
Small	reference	
Medium	1.51(0.28-8.16)	0.63
Large	4.31(0.99-18.77)	0.05
**Hospital Location and Teaching Status**		
Rural	reference	
Urban non-teaching	0.86(0.09-7.97)	0.9
Urban teaching	3.32(0.43-25.68)	0.25

## Discussion

Frequent and prolonged hospitalizations continue to represent a significant burden to individual patients regarding quality of life and on healthcare systems financially. It has been well-established that racial/ethnic disparities in hospitalization rates exist for various diseases, including multiple myeloma.^6,9^ Currently, limited data exist on the relationship between race/ethnicity and hospitalization rates in plasma cell leukemia. However, prior studies have demonstrated no significant differences for race in overall survival (OS) or causes of death for PCL.^10^ Overall, the rate of hospitalizations for PCL tended to decrease over the past decade across all groups, possibly owing to changes in the standard of care to more outpatient-based regimens and overall better outcomes for patients with MM.^1^

In our study, no significant associations between hospitalization rates in patients with PCL were seen for either race/ethnicity or other sociodemographic factors, suggesting the possibility that the same factors driving increased hospitalization rates in MM may not be present to the same degree in PCL. In addition, hospitalization rates in PCL did not correlate with mortality within this cohort. The overall lower incidence of PCL, more significant disease burden, and poor prognosis across all groups may contribute to these findings.^6,10^ This is supported by a recent study using the Surveillance Epidemiology and End Results (SEER) database, which demonstrated that PCL had a higher mortality rate within 6 months compared to MM, lower median OS, and in contrast to MM, neither age at diagnosis nor race was associated with prognosis in PCL.^11^ It has been increasingly clear that PCL has distinct biology from MM. For example, there are cytogenetic differences in PCL compared with MM, such as a higher prevalence of 17p deletions, hypodiploidy, and translocation t(14;16), as well as clinical features such as higher incidence of cytopenias, elevated lactate dehydrogenase (LDH), and extramedullary involvement.^12,13^ It is currently unclear whether there is a relationship between race/ethnicity and these molecular and clinical differences in PCL specifically.

Our analysis has some limitations. The use of the NIS database includes the fact that NIS does not identify or track individual patients, and thus does not differentiate between first-time versus recurrent hospitalizations, and also does not capture observation stays. Our data correspond to hospitalizations, not individual patients, and one patient can be hospitalized several times in different situations. NIS identifies diseases based on ICD codes and may not fully reflect diagnostic accuracy.^14^ It is essential to acknowledge that the diagnostic criteria of PCL have varied and may remain variable over time. Moreover, some patients with PCL may die outside of the hospital, and this type of death could vary across racial populations, which may have affected our results.

## Conclusions

This study found no significant difference between racial groups in hospitalizations and/or hospitalization-related mortality for patients with PCL. With increasing evidence that PCL is cytogenetically distinct from MM,^15^ more investigation into biological and sociodemographic factors that affect healthcare utilization and treatment outcomes should be carried out.

### Abbreviations

MM: multiple myeloma; PCL: plasma cell leukemia; ICD-CM: International Classification of Disease Clinical Modification; NIS: national inpatient sample; LDH: lactate dehydrogenase; SEER: Surveillance Epidemiology and End Results; OS: overall survival; OR: odds ratio; CI: confidence interval; NH: non-Hispanic; AAPC: Average Annual Percentage Change; CAR-T: chimeric antigen receptor T cell; NHANES: National Health and Nutritional Examination Survey; MGUS: monoclonal gammopathy of undetermined significance.

### Ethics approval and consent to participate

Given that the data is publicly available, the Institution Review Board at the University of Arkansas for Medical Sciences considered the study exempt.

### Consent for publication

We give the Publisher permission to publish the Work.

### Availability of data and material

Data used in this study are publicly available.

### Competing interests

The authors have no relevant financial or non-financial interests to disclose.

### CRediT - Authors’ Contribution

Writing – original draft: Cindy Wu (Equal), Samer Al Hadidi (Equal). Writing – review & editing: Cindy Wu (Equal), Deepa Dongarwar (Equal), Samer Al Hadidi (Equal). Data curation: Deepa Dongarwar (Lead). Formal Analysis: Deepa Dongarwar (Lead). Methodology: Deepa Dongarwar (Lead). Software: Deepa Dongarwar (Lead). Conceptualization: Samer Al Hadidi (Lead). Supervision: Samer Al Hadidi (Lead).
